# Rear Admiral John Yeatman Taylor, U.S.N.

**Published:** 1911-12

**Authors:** 


Rear Admiral John Yeatman Taylor, U.S.N., retired, formerly Medical Director, committed suicide in Washington, November 16, 1911. He was 82 years old, a graduate of Jefferson, 1852, and had seen service with Farragut during the Civil War. His death was attributed to the killing of his son by an automobile, two years ago.

